# Economic performance and cost-effectiveness of using a DEC-salt social enterprise for eliminating the major neglected tropical disease, lymphatic filariasis

**DOI:** 10.1371/journal.pntd.0007094

**Published:** 2019-07-01

**Authors:** Swarnali Sharma, Morgan E. Smith, James Reimer, David B. O’Brien, Jean M. Brissau, Marie C. Donahue, Clarence E. Carter, Edwin Michael

**Affiliations:** 1 Department of Biological Sciences, University of Notre Dame, Galvin Life Science Center, Notre Dame, IN, United States of America; 2 Grosse Pointe Park, MI, United States of America; 3 Chattahoochee Hills, GA United States of America; 4 College of Science, University of Notre Dame, Notre Dame, IN, United States of America; 5 Eck Institute of Global Health, University of Notre Dame, Notre Dame, IN, United States of America; RTI International, UNITED STATES

## Abstract

**Background:**

Salt fortified with the drug, diethylcarbamazine (DEC), and introduced into a competitive market has the potential to overcome the obstacles associated with tablet-based Lymphatic Filariasis (LF) elimination programs. Questions remain, however, regarding the economic viability, production capacity, and effectiveness of this strategy as a sustainable means to bring about LF elimination in resource poor settings.

**Methodology and principal findings:**

We evaluated the performance and effectiveness of a novel social enterprise-based approach developed and tested in Léogâne, Haiti, as a strategy to sustainably and cost-efficiently distribute DEC-medicated salt into a competitive market at quantities sufficient to bring about the elimination of LF. We undertook a cost-revenue analysis to evaluate the production capability and financial feasibility of the developed DEC salt social enterprise, and a modeling study centered on applying a dynamic mathematical model localized to reflect local LF transmission dynamics to evaluate the cost-effectiveness of using this intervention versus standard annual Mass Drug Administration (MDA) for eliminating LF in Léogâne. We show that the salt enterprise because of its mixed product business strategy may have already reached the production capacity for delivering sufficient quantities of edible DEC-medicated salt to bring about LF transmission in the Léogâne study setting. Due to increasing revenues obtained from the sale of DEC salt over time, expansion of its delivery in the population, and greater cumulative impact on the survival of worms leading to shorter timelines to extinction, this strategy could also represent a significantly more cost-effective option than annual DEC tablet-based MDA for accomplishing LF elimination.

**Significance:**

A social enterprise approach can offer an innovative market-based strategy by which edible salt fortified with DEC could be distributed to communities both on a financially sustainable basis and at sufficient quantity to eliminate LF. Deployment of similarly fashioned intervention strategies would improve current efforts to successfully accomplish the goal of LF elimination, particularly in difficult-to-control settings.

## Author summary

With less than three years remaining for meeting the initial 2020 target set by WHO for accomplishing the global elimination of Lymphatic Filariasis (LF), concerns are emerging regarding the feasibility of meeting this goal using the current tablet-based Mass Drug Administration strategy. Salt fortified with the antifilarial drug, diethylcarbamazine (DEC), could offer an intervention that avoids many of the barriers connected with tablet-based elimination programs. We analyzed the economic performance and cost-effectiveness of a novel DEC-salt social enterprise developed and tested in Léogâne arrondissement, Haiti, as a particularly significant strategy for accomplishing sustainable LF elimination in such complex settings. We show that because of increasing revenue from the sale of the DEC salt over time, expansion of its delivery in the population, and the adverse effect of continuous consumption of the drug on worms, the delivery of DEC through a salt enterprise can represent a significantly more cost-effective option than annual DEC tablet-based MDA for accomplishing LF elimination in settings, like Léogâne. We indicate that development of policy and research into how to deploy similarly-fashioned interventions, or work with the salt industry to increase population use of medicated salt, would improve present efforts to successfully accomplish the elimination of LF.

## Introduction

Lymphatic filariasis (LF), a mosquito-borne neglected tropical disease (NTD), commonly known as elephantiasis, is one of only parasitic six diseases currently targeted for potential global eradication by 2020 using preventive mass chemotherapy [1–3]. Despite the impressive expansion of a WHO-led elimination program aimed toward the meeting of this goal in all endemic countries since 2000, stakeholders committed to global LF elimination have recognized that the current tablet-based mass drug intervention is resource-intensive, can face significant compliance issues with time, and may be difficult to implement in remote or socio-ecologically complex areas, such as urban and socio-politically unstable settings, hampering foreseen elimination goals [4–8]. These difficulties have heightened interest in investigating the impacts of either approaches aimed at scaling-up treatment strategies or inclusion of preventive activities into drug programs (such as supplemental vector control), or evaluation of novel intervention technologies, that can effectively overcome current barriers in order to accelerate parasite elimination [9–11].

Salt fortified with the anti-filarial drug, diethylcarbamazine (DEC), could offer an intervention that avoids many of the above issues connected with tablet-based elimination programs [12–18]. Indeed, DEC-salt has played a major role in the elimination of LF in a number of pilot and region-wide settings in Africa, Central America, and Asia [5,12–17]. The low dose of DEC (0.1–0.6% [w/w]) used in these studies and programs was well tolerated and rarely associated with adverse reactions. It also has the potential to be more effective than tablet-based Mass Drug Administration (MDA) programs via reduction of the durations of intervention required to interrupt parasite transmission [18]. Moreover, fortified salt can also be provided to a population without developing a dedicated public distribution system, overcoming the need for developing an effective health infrastructure capable of distributing anti-filarial drugs at the high coverages needed for achieving elimination [5,7,8].

Haiti is one of only four countries remaining in the Americas where LF is still endemic [8,19]. MDA using DEC first started in the country under the National Program to Eliminate LF (NPELF) in 2000, and by 2005 had expanded to mass treatment of some 1.6 million people at least once in 24 of the initially endemic 120 communes of the country ([8]. However, following funding, sociopolitical, and natural disaster-based challenges to further scaling up, the program realized full national coverage of all the endemic communes in the country only by 2012 [5,7,8,20]. This delay together with the technical challenge of interrupting transmission in areas of highest prevalence even with high levels of coverage using the suggested five successive years of annual MDA [5,7,8,20], indicate that it is unlikely that NPELF will meet the goal of accomplishing LF elimination in the country by the target year of 2020. In 2006, partly to overcome the above issues, a project was initiated with the collaboration of Congregation de Sainte Croix, the Notre Dame Haiti Program, and the Ministry of Public Health and the Population (MSPP), focused on the local processing and marketing of DEC-mediated salt co-fortified with potassium iodate as an alternative means to facilitate the elimination of LF (and prevention of iodine deficiency disorders) in Haiti [8]. Based on the principle of employing a social enterprise framework for providing goods and services in an entrepreneurial and innovative fashion to solve social problems [21–23], this project purchases both local and imported raw salt which are then cleaned, sized, fortified and packaged for sale in the local market as food-grade co-fortified salt, raising the potential of using a business-based approach to delivering DEC-medicated salt as a sustainable means to accomplish LF elimination in settings, such as Haiti.

Here, our major aim was to examine the economic performance and effectiveness of using the Haitian social enterprise-based framework for producing and marketing DEC-fortified salt as a sustainable, cost-effective, model for achieving the long-term elimination of LF, focusing on Léogâne arrondissement, Haiti. A cost-revenue analysis combined with a mathematical modeling-based evaluation of the cost-effectiveness of the DEC salt social enterprise compared to standard MDA was carried out to undertake this analysis. Specifically, we evaluated the economic performance and social value of the enterprise by assessing: 1) the growth in salt production, costs of resources consumed, and revenues from sales gained to determine break-even points, 2) the impact of the product-mix used for realizing the socially-relevant sale price of the salt, and 3) its cost-effectiveness compared to tablet-based MDA for accomplishing LF elimination in the study setting of Léogâne arrondissement, Haiti. We discuss the results in terms of how using a social enterprise can offer a sustainable and innovative strategy for accomplishing LF elimination in Haiti, and similarly resource-constrained settings, that face both programmatic and social difficulties in delivering long-term tablet-based LF MDAs.

## Methods

### Overview

We carried out a cost-revenue analysis to evaluate the production capability and financial feasibility of the developed DEC salt social enterprise via assessment of the relationship between fixed and variable costs versus the revenue received [24–27] and a modeling study centered on applying a dynamic transmission model to evaluate the cost-effectiveness of using this intervention versus standard annual MDA for eliminating LF in Léogâne arrondissement, where the salt enterprise operates [28–31]. The cost-revenue analysis was based on costs and revenue data contained in financial accounts during the production phase of the salt enterprise from 2013 to 2018, while the break-even analysis was carried out over a time horizon that ranged between 2013 to the year when the break-even point was attained. The predicted timelines, in months or years, to LF elimination along with the costs of annual MDA versus the net cost of supplying DEC-fortified salt until elimination was achieved were used to carry out the cost-effectiveness modeling study.

### Cost-revenue and break-even analyses

#### Cost estimation

From the operational perspective, these included periodic financial investments primarily used for purchasing large equipment; fixed costs (administrative, marketing, maintenance, and leasing costs) and variable costs (costs of raw salt, salt packaging, custom fees, labor, transportation, and drug supplies). As the salt enterprise used a product-mix business strategy of producing three different types of salt to meet demands and application in different market segments, viz. industrial–untreated salt processed to meet the requirements of various industrial applications, coarse and fine single-fortified salt (treated with 40 ppm of potassium iodate only) and double-fortified salt (treated with both 40 ppm of potassium iodate and 0.32% DEC [w/w]), to assure price competitiveness of the double-fortified salt with the local table salt in the market and to meet customer preference for either coarse or fine edible salt, we additionally estimated the variable costs of the two fortified salt types. At the project level, we multiplied the unit cost with the unit quantity of each cost item consumed on a yearly basis to obtain the annual total cost of producing all three types of salt.

#### Revenue estimation

The unit sale price was multiplied by the unit quantity sold per year to quantify the annual revenue obtained by the sale of each salt type. Total revenue was simply the sum of revenues obtained from the sale of each salt type.

#### Forecasting break-even time points

This was conducted by projecting forward the total costs (investment + fixed + variable costs) and total revenues from the sale of all three types of salt estimated for different periods between 2013 to 2018 until the point at which cash flow from the project (total revenue–total cost) becomes zero or the break-even for the enterprise is attained [24–27]. Simple linear forward projections were used to make these calculations. Costs and revenues (both in US$) were used undiscounted in this study.

### Cost-effectiveness modeling

#### Overview of the LF transmission model

We extended the data-driven Monte Carlo population-based EPIFIL model for predicting local LF infections [32–36] to include comparative costs and simulations of the effectiveness of the standard two-drug (DEC plus Albendazole (ALB)) tablet-based MDA used in Haiti versus consumption of double-fortified salt sold by the salt enterprise to perform this analysis. We used a data-model assimilation technique based on the Bayesian Melding (BM) algorithm to calibrate the LF model to the microfilariae (mf) prevalence data observed in our Léogâne endemic setting [28–31], and used the localized model to simulate the impact of either intervention on timelines required to decrease the community-level mf prevalence below the WHO-mandated elimination threshold of 1% mf [1,2]. Modeling of the impacts of MDA using DEC+ALB and DEC-medicated salt followed our previous methods published in detail in Smith et al [18].

#### Input epidemiological data

The data sources used for calibrating the LF model were collected from Léogâne commune, Haiti [28–31]. The epidemiological data inputs encompassed information on baseline community-level mf prevalence (15.5%), the annual biting rate (ABR) estimated inversely by model fitting to mf prevalence data [37], and details regarding the dominant mosquito genus (*Culex quinquefasciatus)*. Published details of the MDA interventions, including the relevant drug regimen, carried out in this setting during 2000–2008 were also assembled and used as required [20,28–31]. All model parameters, functions, and fitting procedures specific to this work are given in detail in S1 Supporting Information.

#### Cost-effectiveness analysis

This was carried out by comparing the costs and effectiveness achieved by the production and sale of DEC-fortified salt versus implementation of the annual tablet-based DEC/ALB MDA for accomplishing LF elimination in Léogâne (as measured by the times by which the initially observed baseline pre-control mf prevalence in this setting reach below the WHO-recommended 1% mf prevalence threshold [1,2]). Timelines in terms of the number of months and years taken by each intervention to reduce the initial prevalence to below the 1% mf threshold were quantified by simulating trends in infection prevalence due to these interventions using the Monte Carlo-based EPIFIL model localized to reflect LF transmission in Léogâne as described above. The impact of the annual tablet-based MDAs was studied by running the model at 65% and 80% population coverages, while we simulated the effect of the produced DEC salt using 65%, 80% and the actual population coverages.

The direct cost for carrying out annual MDA was fixed conservatively at US$0.64 per person inclusive of drug cost, given the finding that this represented the average cost of treating an individual in Haiti once initial costs stabilized [29,38], while the cost of delivering DEC-medicated salt per person per year was estimated from the difference between the costs of production and the revenues gained from sales of the salt. Simulations of cost-effectiveness were carried out by fixing the Léogâne arrondissement population at 500,000 [39]. All costs are calculated from the perspective of the direct financial costs of delivering either DEC tablets or fortified salt (ie. not inclusive of any opportunity costs that may be involved in delivering these strategies) by the health service or program provider.

## Results

Costs and production of salt

Table 1 summarizes the investment, fixed and variable costs incurred in establishing and operating the Léogâne DEC salt enterprise. These costs are presented for the years between 2013–2018 when production of food-grade fortified salt began (following an experimental phase which addressed technical issues in the fortifying of salt with DEC) along with corresponding data on the quantity of the three different types of salt (industrial, coarse and fine single-fortified, coarse and fine double-fortified) produced and sold. Note that investments occurred periodically during different expansion phases (2013, 2014, and 2017), and were primarily used to acquire capital items either from the US or from within Haiti. These were recorded as fixed assets, and comprised factory items, such as different types of pumps, screens, control systems, hoppers, sealers, storage tanks, generators, and office equipment. Fixed costs, i.e., costs that remain the same whatever the level of output produced or products sold, included operating expenses, while variable costs comprised costs of items which scaled with production volume [24,25]. Examples of components of the latter two cost types incurred are given in Methods.

**Table 1:** Cost (investment, fixed cost and variable cost (in US$)) and summary of all types of salt (industrial salt, coarse and fine single-fortified salt, and coarse and fine double-fortified salt) produced (tons) from 2013–2018.

Year	Cost (US$)			Salt Production (Tons)				
	Investment	Fixed Cost	Variable Cost	Industrial	Single-fortified		Double-fortified	
					Coarse	Fine	Coarse	Fine
2013 (Jan-Dec)	98,418	36,612	75,176	10	0	0	230	0
2014 (Jan-Dec)	397,249	180,589	120,296	272	1	5	470	0
2015 (Jan-Dec)	0	275,495	225,367	557	146	33	334	7
2016 (Jan-Dec)	0	275,495	392,882	2140	452	206	479	61
2017 (Jan-Dec)	49,280	558,904	601,308	2811	648	308	979	91
2018 (Jan-Dec)	23,000	352,000	800,000	2431	1013	527	1841	33

The data on salt production show that initial output was low (e.g., 10 metric tons of industrial salt and 230 tons of coarse double-fortified salt in 2013, compared to 272, 6, and 470 tons of industrial, single-fortified, and double-fortified salt respectively in 2014). To increase production, a three phase expansion of the project was introduced beginning in the year 2013. Phase 1 focused on installing higher capacity processing equipment (January 2013-March 2014), while Phase 2 aimed to expand processing capacity by adding bulk storage and extension of the brine-washing system (May 2014-March 2015), and Phase 3 further expanded these washing, processing and storage capacities (January 2017-June 2018). These expansion phases meant that both the fixed and variable costs of salt production varied over time (Table 1), but in general, and as expected, as production expanded the variable costs increased proportionately while the fixed costs simply reflected increases in capital investments. By contrast, the production figures show that the manufacture and sale of each type of salt increased steadily, with industrial salt dominating production particularly toward the later years followed by double-fortified (i.e., DEC and iodine medicated) and single-fortified (iodine-medicated only) coarse salts. Given the population preference for coarse edible salt in Léogâne (and Haiti in general), fine salt production and sale lagged behind in volume (Table 1). The figures, however, demonstrate that with expansion in capacity the enterprise was able to achieve significant production volumes for the double-fortified salt by year 2018 (1841 metric tons recorded for the coarse variety).

### Net cash flow and break-even forecasting

**Fig 1:**
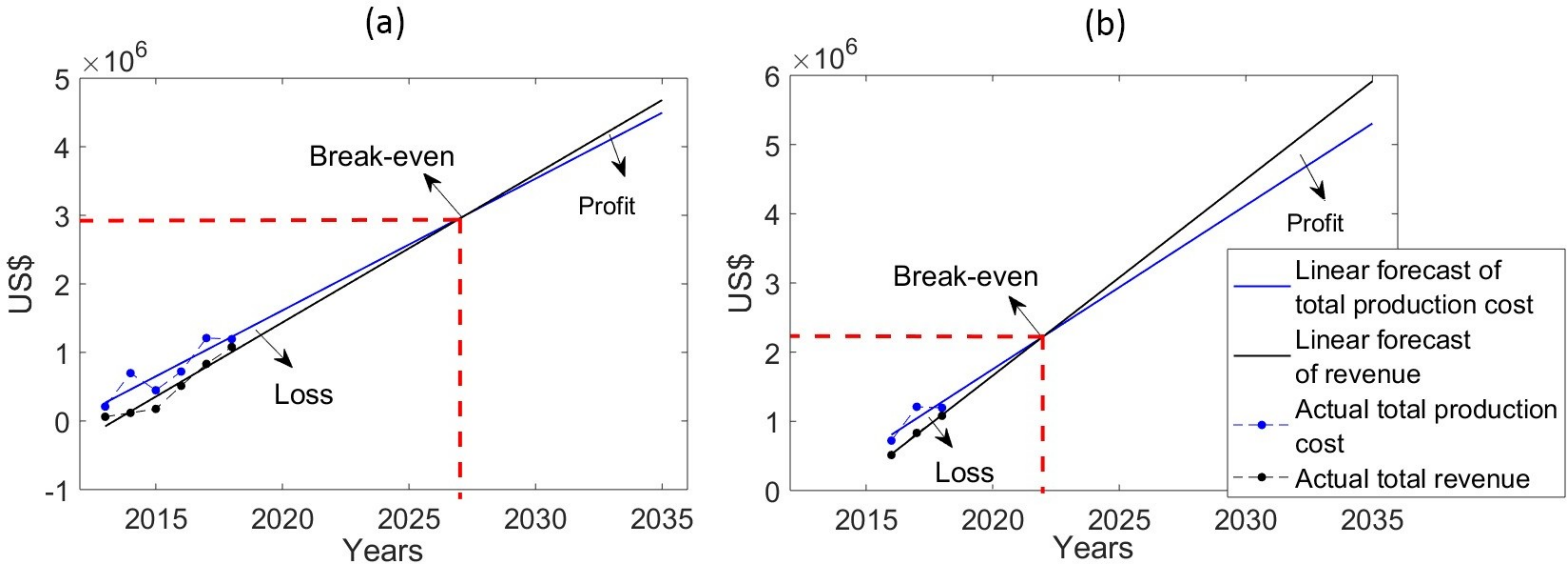
Forecasts of the break-even years of the salt enterprise. Results are shown from using a simple linear model to project forward the total project costs and revenues (in US$). **(a)** Forecast of break-even year using total cost and total revenue data calculated for 2013–2018 given in Table 2. **(b)** Forecast of break-even year using total cost and total revenue data calculated for 2016–2018 given in Table 2. The red dotted line indicates the break-even point for each scenario.

The total annual costs of salt production (= Investment + Fixed Costs + Variable Costs) and revenues attained from the sale of all three types of salt are given in Table 2. Total annual costs increased as production expanded (Table 1) from US$210,206 in 2013 to US$1,175,000 in 2018 –i.e., approximately five times–but the figures show that revenue increased even faster, up to 17 times that obtained initially in 2013 (US$61,636) to close to cost of production by 2018 (US$1,064,000). The net cash flow [24–27] figures in the Table, which represent the difference between total production cost and total revenue, although being negative for all the years from 2013–2018, capture this increasing revenue returns (as reflected by the declining negative trend in the net cash flow) towards the later years, suggesting that the project is close to achieving break-even or profitability in the near future. To estimate the exact time point when the enterprise is likely to break-even (i.e., the time point when the total program cost is equal to the total revenue), we employed a simple linear model to project forward the total project costs and revenues calculated for 2013–2018. Fig 1(A) shows that if we use the full data on annual costs and revenues obtained for the whole 2013–2018 period, the project will break-even in 2027. However, if we use the data from 2016–2018, when total salt production had reached significant levels (Table 1), to predict the time point at which the break-even point will be achieved, this will occur earlier by year 2022 (Fig 1(B)).

**Table 2:** Year wise details of total cost, total revenue and net cash flow attained by the Haiti salt enterprise between 2013 to 2018. The total costs shown are calculated using the cost data for the production of all three types of salt, while total revenue is calculated using the revenue data from the sale of all three types of salt for each year.

Year	Total Cost (US$)	Total Revenue (US$)	Net Cash Flow
**2013**	210,206	61,636	-148,570
**2014**	698,134	118,583	-579,551
**2015**	448,168	175,629	-272,539
**2016**	721,070	512,588	-208,482
**2017**	1,209,492	832,285	-377,207
**2018**	1,175,000	1,064,000	-111,000

**Fig 2:**
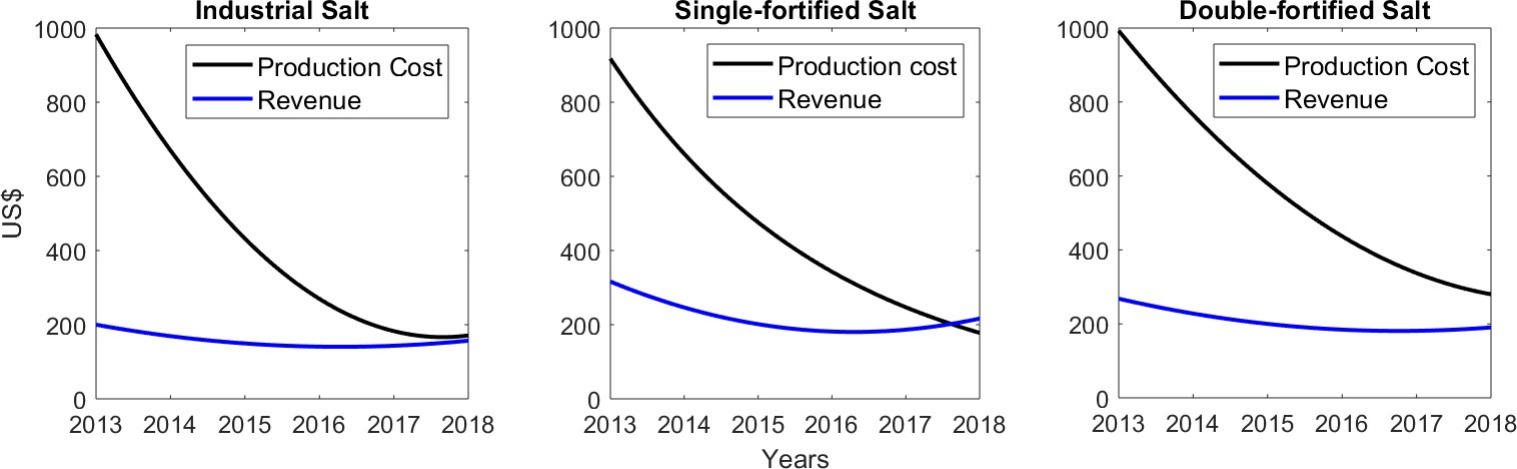
Revenue and production cost for industrial, single-fortified and double-fortified salt. Both revenue and production cost (in US$) for each salt are shown per ton.

Fig 2 depicts the per ton revenues from sales and costs of producing the three different types of salts for the years 2013–2018. The results show that for all salt types, while initially there was a large difference between the production cost and revenue per ton (i.e., a large negative cash-flow), this difference decreased for each salt category with time. This occurred faster, however, in the case of the industrial and single-fortified salt, such that break-even was achieved by 2018. By contrast, the cash-flow from the production and sale of the double-fortified salt was still negative (lower revenue compared to production costs) at the end of the present study period of 2018. This result shows, first, that the overall break-even estimated in this study (Fig 1) for the project is due to the delay in reaching the break-even year for double-fortified salt. Second, it also highlights how the mixed product strategy of producing different types of salt targeting different market sectors can allow the more profitable products (industrial, single-fortified salt) to subsidize the sale of a product (double-fortified salt) whose cost (approximately US$200 per ton (Fig 2)) needs to be kept competitive with other edible salt sold (retailed at $US265 per ton in Haiti) in the local market.

### Marginal cost of manufacturing DEC-medicated salt

This was evaluated by analysis of the difference in the variable costs of producing the single-fortified (which included only potassium iodate) versus the double-fortified (both potassium iodate and DEC included) salt types, given that the investment and fixed costs going into manufacturing all salt types were shared equally between each type. The variable costs per ton for producing the single-fortified (coarse and fine) and double-fortified (coarse and fine) salt for two types of bags/bales (25.0-kg bags and 12.5-kg bales) are provided in Table 3 for the years 2014–2018 when both salt products were produced (note that production of single-fortified salt began only in 2014 (Table 1)). Analysis of the difference in the variable costs for producing the single-fortified versus the double-fortified salt indicate that this was consistently about US$70 per metric ton, irrespective of which type—coarse or fine variety—or types of bags/bales were produced (Table 3). Given that the price of DEC, as delivered by Syntholab Chemicals to the project was US$21.60 per kg (James Reimer, personal communication), and DEC salt in this project was fortified with 0.32% DEC by weight or with 3.2kg of DEC/ton, it can be seen that the cost of producing one metric ton of DEC salt works to be US$ 69.12/ton (i.e., 3.2 kg DEC x US$21.60 per kg). This result indicates that the marginal cost, or difference in the variable cost, of adding DEC to single-fortified salt (US$70; Table 3) was simply due to the purchase price of DEC.

**Table 3:** Marginal cost of manufacturing DEC-medicated salt. This was evaluated by analysis of the difference in the variable costs of producing the single-fortified (which included only potassium iodate) versus the double-fortified (both potassium iodate and DEC included) salt types, given that the investment and fixed costs going into manufacturing all salt types were shared equally between each type.

Year	Types of Bags/Bales	Variable cost of Coarse Salt (US$/Ton)			Variable cost of Fine Salt (US$/Ton)		
		Single- fortified	Double- fortified	Difference	Single- fortified	Double- fortified	Difference
2014	25.0-kg Bags	150	220	70	160	230	70
	12.5-kg Bales	190	260	70	200	270	70
2015	25.0-kg Bags	145	215	70	155	225	70
	12.5-kg Bales	185	255	70	195	265	70
2016	25.0-kg Bags	145	215	70	155	225	70
	12.5-kg Bales	185	255	70	195	165	70
2017	25.0-kg Bags	135	205	70	145	215	70
	12.5-kg Bales	175	245	70	185	255	70
2018	25.0-kg Bags	135	205	70	145	215	70
	12.5-kg Bales	175	245	70	185	255	70

### Population coverage attained by the DEC salt enterprise

Table 4 shows the potential increase in demand for salt in Léogâne arrondissement calculated as a function of changes in population size from 2013 to 2018. It also presents the potential DEC-fortified salt coverage which may be achieved in the setting by increasing sale of the double-fortified salt produced over this period. The annual population size estimates from 2013 onwards were predicted using a growth rate of 1.28% [39], whereas the yearly population demand for edible salt was calculated by assuming that the daily salt consumption per person is 15gm (average of the reported daily per-capita consumption in Haiti of 10gm and 19gm [5,40]). Assuming that the sale of double-fortified salt is widespread in the community (i.e., not targeted towards one segment of the population), coverage of DEC salt in Léogâne can then be roughly estimated simply by dividing the quantity of salt produced over the estimated demand. The results from this calculation, listed in the last column of Table 4, shows that as production increased rapidly from 2013 to 2018, this would increase potential population coverage achieved by sales of the DEC-medicated salt from as low as 8.45% in 2013 to approximately 65% in 2018.

**Table 4:** Details of production of double-fortified (DEC-medicated) salt (tons), its potential demand/year, and coverage in Léogâne Arrondissement.

Year	Quantity of double-fortified (DEC-medicated) salt (Tons)	Population of Léogâne Arrondissement*	Potential Demand of salt/year (Tons)**	Coverage (%)
**2013**	230	497,274	2,723	8.45
**2014**	470	503,241	2,755	17.06
**2015**	341	509,280	2,788	12.23
**2016**	540	515,799	2,824	19.12
**2017**	1070	522,401	2,860	37.41
**2018**	1874	529,088	2,897	64.69

*Population growth rate of Haiti is 1.28% [39]

**Assuming that the salt consumption rate per person per day is 15gm [5,40]

### Cost-effectiveness modeling

**Fig 3:**
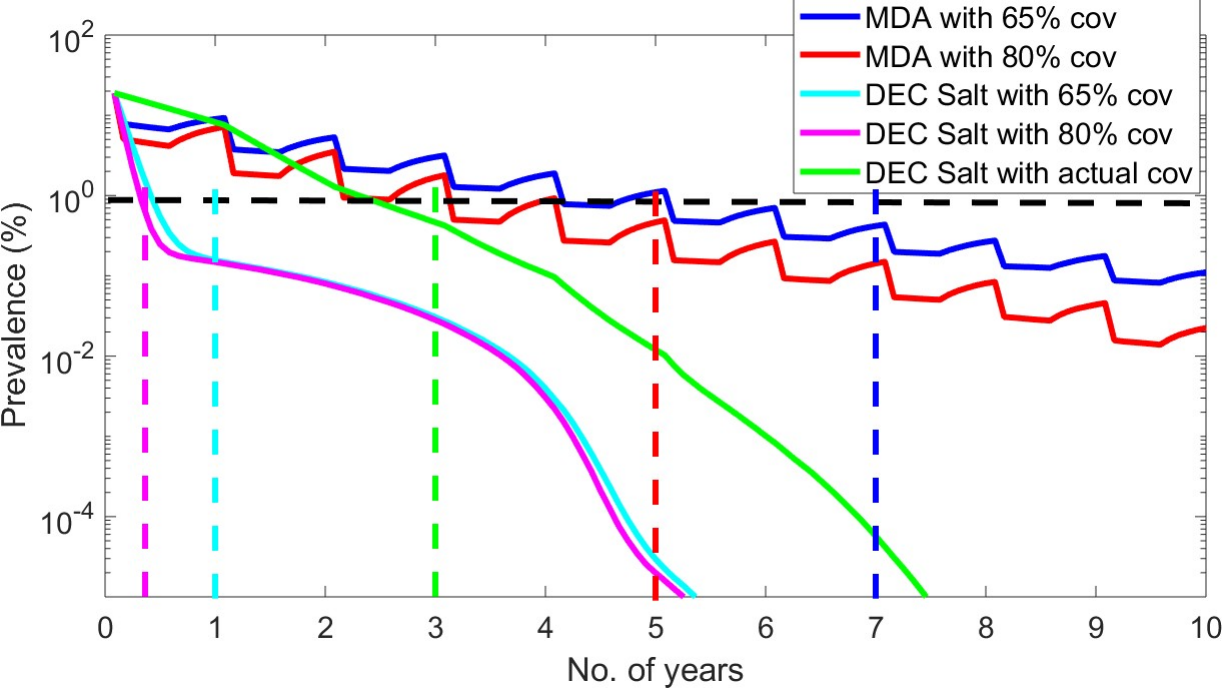
Timelines to reach the WHO recommended 1% mf threshold for MDA implanted at 65% and 80% coverage and DEC-medicated salt at 65%, 80%, and actual population coverages. The median model predicted trajectory is shown for each intervention strategy. The black dotted horizontal line indicates the WHO recommended 1% mf prevalence threshold. The blue, red, sky blue, purple and green dotted vertical lines indicate the time points to reach 1% mf threshold using either MDA at 65% and 80% coverages or DEC Salt at 65%, 80%, and actual coverage, respectively.

Fig 3 portrays the predicted timelines to LF elimination in Léogâne under each of the MDA and salt interventions investigated. For MDA, the depicted simulations indicate that it would take up to 84 months at 65% coverage, and 60 months at 80% coverage, respectively, to reach the 1% mf threshold. By contrast, the model predictions show that it will take just 1 year (12 months) at 65% coverage, 5 months at 80% coverage, and 3 years or 36 months if actual population coverage (Table 4) is used to reduce the pre-control prevalence to below this threshold via consumption of the traded DEC salt (Fig 3). This highlights the dramatic effect that daily consumption of DEC-medicated salt even at low dosages (0.32% w/w) would have compared to annual intake of higher dosages of DEC (and ALB) as provided by tablet-based MDA for eliminating LF infection in an endemic setting [12,15–18]. Note that the actual DEC salt population coverages (Table 4) used in this analysis assumed that salt supply occurred uniformly and sale was restricted to Léogâne arrondissement only. Any changes in these parameters would mean attaining lower annual population coverages than shown in Table 4; however, a sensitivity analysis using coverage values 15–20% lower than those depicted in the Table did not affect the above timelines significantly.

The comparative costs of carrying out annual MDA versus supplying DEC-medicated salt are shown in Table 5. These show that while the total cost of delivering annual MDA (here fixed at US$0.64 per person, inclusive of the cost of the drug [29,38]), simply scaled with population growth, and will continue to be substantial on a yearly basis until LF elimination is achieved, the net cost of supplying DEC-fortified salt through the social business model will decline dramatically with time as production costs decrease and revenues begin to increase over time (Fig 2). Indeed, projection forward of the net or revenue—production cost data collected during the years 2013–2018 indicates that the total and per capita net costs of DEC salt provided through the present social enterprise could potentially even become zero at the time point (2027) when the project breaks even (Fig 1(A)).

**Table 5:** Forecast of the population of Léogâne, annual cost of supplying double- fortified (DEC-medicated) salt per person per year, and total costs of treatment with DEC-medicated salt and MDA respectively for the years 2013–2027. The population growth rate for Haiti estimated in year 2015 (1.28%) was used in making the population size estimations shown in the Table, whereas a simple linear model based on the cost and revenue data connected with DEC-medicated salt production and sale (both fine- and coarse salt types) for the years 2013–2018 (Fig 2) was used to forecast the annual cost of salt-based DEC treatments per person and for the total population between 2019–2027.

Year	Population of Léogâne*	Annual Cost for Double fortified (DEC-medicated) salt per person (US$)	Total cost for treating with DEC-medicated salt (US$)	Total cost for MDA (US$)**
**2013**	497,274	3.38	1,680,786	318,255
**2014**	503,241	4.27	2,148,839	322,074
**2015**	509,280	0.94	478,723	325,939
**2016**	515,799	0.97	500,325	330,111
**2017**	522,401	1.01	527,625	334,337
**2018**	529,088	0.58	306,871	338,616
**2019**	535,860	0.61	329,018	342,950
**2020**	542,719	0.51	276,787	347,340
**2021**	549,666	0.41	223,164	351,786
**2022**	556,702	0.30	168,124	356,289
**2023**	563,827	0.20	111,638	360,849
**2024**	571,044	0.09	53,678	365,468
**2025**	578,353	0.01	5,783	370,146
**2026**	585,757	0.001	586	374,884
**2027**	593,254	0	0	379,683

*Population growth rate of Haiti is 1.28% [39]

**Using cost for annual MDA as US$0.64 per person/year [38]

We used the total costs of implementing each strategy until LF elimination (crossing below the 1% mf threshold) is achieved to investigate the cost effectiveness of either strategy. The total costs and effectiveness of using MDA at 65% and 80% coverages and supplying DEC salt at 65%, 80% and the actual coverages given in Table 4, were evaluated and compared via calculations of the average and incremental cost effectiveness ratios [10,41–47]. With respect to DEC salt, we also conducted the analysis using three different net costs of salt production per person per year as recorded in Table 5: (i) average of the net cost of salt produced per person calculated for the years 2018–2020, i.e., US$0.57 per person/year, (ii) average of the net cost of salt per person for the years 2021–2023, i.e., US$0.3 per person/year, and (iii) average of the net cost of salt per person for the years 2025–2027, i.e., US$0.025 per person/year. This was performed to assess the sensitivity of the present results to changes in the steeply declining net cost of salt production over time observed in this study.

The results from this exercise are shown in Table 6, and indicate, principally, that irrespective of coverage, the costs of using MDA are significantly greater than those arising from using the salt strategies, primarily because of its lesser effectiveness as well as higher and stationary unit cost. Among the three salt scenarios, costs for eliminating LF, as expected, declined with decreasing net cost of production over time with scenario three showing the lowest costs. However, for all strategies, while the most effective strategy is to deliver MDA or salt at 80% coverage, the most cost-effective option (in terms of the incremental cost-effectiveness ratio (ICER)) also occurred at this coverage level for both MDA and the DEC salt strategies with incremental costs of either of these options being negative and incremental effects positive over their corresponding next-effective alternative (ICER: US$88,000 per intervention year saved by the 80% MDA strategy compared to an average CER of US$17,333 for carrying out 65% MDA using the cost of implementing annual MDA fixed at US$0.64 per person/year in Léogâne [38], and an additional saving of US$3,876 per intervention year saved by the 80% DEC salt option over the strategy delivering DEC salt at actual recorded coverages when the net cost of delivering medicated salt was fixed at US$0.57 per person (based on durations of interventions in years and total costs given in Table 6)). Note moving from delivering DEC salt at actual recorded coverages to providing salt at 65% coverage results in higher predicted total costs but also a saving of 2 years, and so this strategy is dominated by the strategy that delivers DEC salt at 80% coverage which results in extra reductions in both costs and the time needed to accomplish LF elimination (Table 6). Similar results were also obtained when the other two net costs of producing DEC salt per person were used in carrying out these calculations.

**Table 6:** Comparison of total costs and CE ratios of population treatment using MDA versus supply of DEC-medicated salt for different drug coverages and DEC salt costs.

Treatment Method	Coverage	No. of months (years) to reach 1% mf threshold	Total Cost (US$)	Cost-Effectiveness Ratio (CER)^#^
Annual MDA*	65%	84 (7)	1,456,000	17,333
	80%	60 (5)	1,280,000	21,333
**DEC salt**^**1**^	65%	12 (1)	185,250	15,438
	80%	5 (0.42)	95,760	19,000
	Actual^†^	36 (3)	105,450	2,929
**DEC salt**^**2**^	65%	12 (1)	97,500	8,125
	80%	5 (0.42)	50,400	10,000
	Actual^†^	36 (3)	55,500	1,541
**DEC salt**^**3**^	65%	12 (1)	8,125	677
	80%	5 (0.42)	4,200	833
	Actual^†^	36 (3)	4,625	129

*Using cost for annual MDA as US$0.64 per person [38], and total population of Léogâne arrondissement 500,000 (fixed).

†Actual coverage of DEC-medicated salt given in Table 4.

^#^CER computed as total cost of each strategy divided by the number of months taken to achieve LF elimination

^1^Average of the net cost of DEC salt produced per person calculated for the years 2018–2020 given in Table 5, i.e., US$0.57 per person/year.

^2^Average of the net cost of DEC salt produced per person calculated for the years 2021–2023 given in Table 5, i.e., US$0.3 per person/year.

^3^Average of the net cost of DEC salt produced per person calculated for the years 2025–2027 given in Table 5, i.e., US$0.025 per person/year.

Overall, thus, these results indicate that because of: 1) decreasing net cost of DEC salt production over time, and 2) expansion of its coverage in the population leading to significantly reduced elimination timelines, the delivery of DEC through a salt enterprise may be significantly more cost-effective than annual DEC tablet-based MDA for accomplishing LF elimination in the Léogâne arrondissement setting. It is also to be appreciated that we used a conservative treatment cost of $0.64 per person for modeling the cost-effectiveness of the tablet-based MDA program in this study. This represented a best-case scenario for the MDA program implemented in Léogâne given that the actual drug costs started out higher (US$1.84 over the first 3 MDAs) before approaching the stabilized value used in our analysis as the program became more efficient [29,38]. Indeed, a recent systematic review indicated that MDA program costs can vary substantially between settings, with an average cost that could reach as high as US$1.32 [48]. Use of such values or inclusion of the actual change observed in the per person treatment cost over time in Léogâne in the present analysis would clearly further increase the cost of MDA over that presented in Table 5, which in turn would lead to an even higher cost-effectiveness ratio for MDA compared to those estimated for DEC salt in this study (Table 6). Nonetheless, depending on the cost of producing and delivering DEC-medicated salt, it is readily apparent that the cost savings to a provider of utilizing a social enterprise framework for DEC delivery can be remarkably high. For example, even at the average cost of US$0.907 per person/year (the worst-case scenario using cost data from 2015–2018 (Table 5)), delivery of DEC salt through the current enterprise for eliminating LF in Léogâne is predicted to result in total costs of US$453,386 and US$190,423 for achieving 65% and 80% coverage of the population respectively, which amounts to only 31% and 15% of the corresponding predicted total costs of using annual MDA at these coverages for achieving the same objective in this study location.

## Discussion

In this study, we have undertaken a performance assessment of a novel social enterprise, developed through a collaboration between Haitian and international partners engaged with LF control in the country, as a means to enhance the delivery of anti-filarial drugs to populations through the trading of salt co-fortified with DEC. Although DEC-fortified salt has been used previously in both pilot and region-wide LF intervention programs in a variety of global regions, ranging from Brazil, Tanzania, India and China to effectively control or eliminate LF [5,12–17], it is to be noted that the developed Haiti salt enterprise is the first attempt anywhere in the world to apply the principles of social entrepreneurship for delivering such an intervention. Recent work has highlighted how such social enterprises–that is, a social mission-driven organization that trades in goods or services for a social purpose–are emerging as a potentially effective supply side solution to the provision of cost-efficient public services in response to government failures, business that seek to extract maximal returns on investment, and unstable non-profit organizations [21–23]. In particular, this work has shown how these business entities can solve social problems via their potential to deliver greater responsiveness, efficiency and cost-effectiveness, through an explicit focus on meeting specific social goals while operating with the financial discipline and innovation of a private-sector business [21–23,49,50].

Although there is continuing debate as to how best to evaluate the performance of social enterprises, it is clear that at least two basic components related to the bottom line of these entities require assessment [21,23]. These primarily include: the economic-financial component for measuring overall organizational efficiency, profitability and hence sustainability, and the social effectiveness of the enterprise [21–23]. Here, we have combined the tools of financial accounting and modeling of cost-effectiveness to measure these components in order to present a first analysis of the utility of using the developed Haitian DEC salt enterprise as a sustainable and economically efficient strategy to bring about LF elimination in programmatically difficult-to-control settings, like the arrondissement of Léogâne, Haiti [10,41–47].

Our analysis of the performance of the present salt enterprise for creating social value first focused on the question of capacity to economically produce sufficient amounts of DEC-fortified salt for significantly affecting the elimination of LF in the study setting. The production figures shown in Table 1 indicate, firstly, that while initial production of all types of salt were low during the initial years of operation, by 2018, and just 5 years after processing began in 2013, the enterprise had reached high levels of both total (5,845 metric tons) and DEC-fortified salt (1,841 metric tons) production, respectively (Table 1). Our analysis of the population coverage that the sale of DEC salt could provide demonstrate that the amounts produced could have potentially resulted in a drug coverage rate of 65% by 2018 (Table 4), which our previous modeling study [18] and the present cost-effectiveness exercise (Fig 3 and Table 6) indicate is sufficient to accelerate the achievement of LF elimination in the Léogâne setting. These findings suggest that as the result of the expansion phases carried out through new capital investments (Table 1), the current DEC salt project may have reached the production capacity required to achieve its stated social mission of using a market-based approach for delivering sufficient edible DEC-medicated salt as a means to bring about efficient LF transmission interruption in the present study setting.

Assessment of the economic and financial performance of the salt enterprise carried out in this study using cost-revenue analysis and financial forecasting has provided further insights regarding the organization efforts used to reach economic equilibrium and hence trading viability. This is an important consideration for evaluating the performance of social enterprises because first and foremost these entities are enterprises, and therefore their social goals can be pursued only by ensuring economic and financial fidelity [21–23]. Our major result in this area is providing clarity regarding how the enterprise’s achieved outputs in salt production and sale may affect its potential to reach break-even points (Fig 1). Specifically, we show that given the observed trends in production costs and revenue to 2018 (Table 2), the present salt enterprise may either break-even by 2027 (if we forecast linearly using all the data from 2013 to 2018), or as early as by 2022 (if we use data collected during 2016–2018 after the project had significantly expanded capacity). This result is clearly dependent on assuming that capacity to produce the increased amount of salt to meet either break-even points is available within the enterprise without any further expansion, and demand for the produced salt in all sectors (industrial to edible salt markets) will also expand commensurately. Nonetheless, the finding that it might be possible to reach the break-even point by 2022 (i.e., over the next 4 years) is encouraging, and suggests that the enterprise is likely to be self-sustaining and could become profitable in the very near future. Indeed, analysis of trends in costs of production and revenues gained per ton of each category of salt (Fig 2) indicate that both the industrial and single-fortified salt categories may already have reached their individual break-even points in 2018, and that the delay for achieving break-even status by the social enterprise is primarily due to the lag experienced by the production and sale of the double-fortified salt. Although the per ton production cost of the latter salt declined as significantly over time as the other two salt categories (Fig 2), indicating the achievement of considerable economics of scale, the need to keep the price of the DEC salt below the marginal cost of adding the drug ($70/metric ton (see Table 3)) to compete with untreated local edible salt in the market means that either: 1) the current market price of the double-fortified DEC salt needs to be revised upwards, or 2) further economies of scale need to be found to bring down production costs, or 3) cross-subsidy from the more profitable categories of salt produced will be required in order to continue with the processing of DEC salt in this setting. While on the one hand, such a capacity to use a product mix strategy innovatively as a means to subsidize the marketing of a product for meeting a social need is a feature of using an enterprise model, note that this may be a particular effect of developing markets in settings, such as Léogâne and Haiti in general, where a strong market-based economy is only just evolving. For other LF endemic settings with stronger market economies and established salt industries, the need for such subsides may be significantly lower meaning that the sale of DEC salt could occur at nearer the true marginal cost of production, i.e., at the actual cost of purchasing the drug itself. Note also that our present forecasts do not fully consider the likely impacts of key swing factors that may significantly affect the profitability of the salt social enterprise, such as enforcement of the 2017 law requiring all food salt in Haiti to be fortified, further progress on market segmentation and the resulting product mix, and significant weather events similar to Hurricane Michael in 2016. Positive changes in the first two factors will clearly enhance the enterprise’s ability to break-even faster and hence attain profitability sooner than predicted in this work.

The cost-effectiveness modeling exercise carried out in this study showed that apart from the efficiency of the business model used for achieving economic and financial sustainability, the salt enterprise may also be more cost-effective than the standard tablet-based annual MDA program for accomplishing LF elimination in Léogâne (Table 6). This is because not only will the population coverage that can be potentially attained by sale of the DEC salt (Table 4) be sufficient to make this strategy more effective than annual MDA in reducing the number of years (3 versus 7 years) required for achieving LF elimination (as defined by reducing mf prevalence below the WHO threshold of 1% [2]), the total overall costs involved—due to both decreasing net cost of production and the need for shorter durations of control—for using the salt approach are also significantly lower than those which will be incurred in running the MDA program. Indeed, this greater social impact of using the present social salt enterprise compared to annual MDA was found to be a general outcome, irrespective of the other intervention coverages investigated (i.e., at 65% coverage–the often normal coverage obtained by MDA programs—or at the recommended optimal coverage of 80% [2]) (Table 6). These results add to our recent modeling work, which highlighted how the continuous consumption of the drug, even at low daily per capita dosages, by resulting in a cumulative impact on the survival of worms and mf which is significantly higher than that afforded by the higher-dosed annual MDA treatment, make DEC medicated interventions, even when delivered at moderate population coverages, a markedly potent strategy for interrupting LF transmission [18]. Finally, an intriguing possibility highlighted by the break-even analysis and the cost forecasting results shown in Fig 1 and Table 5 is that using a social enterprise strategy for delivering DEC through marketing of medicated salt could in principle also lead to zero disease elimination cost for a provider (viz. donor or health system) once the social business attains profitability (i.e., return a positive cash-flow). This is an important result, and demonstrates how using a social enterprise that pursues a social goal by production of services and goods whilst respecting economic efficiency may offer an effective, financially sustainable, intervention strategy in settings facing major fiscal, infrastructural and logistical barriers to carrying out tablet-based programs aiming to control or eliminate parasitic infections.

The present performance evaluation primarily focused on internal (labor, capital, income and taxes) and external (goods and services bought outside the company) expenses/resources related to the economic viability of the salt enterprise [21]. However, estimation of the full social value of a sustainable health social enterprise must also consider, apart from the social benefits accruing from reducing disease only, the wider consequences for a community [21]. Benefits here could be via the choice and use of resources that further address the community interest, such as choosing local salt suppliers to favor short supply chains, choosing socially certified suppliers, adopting a regime of decent work conditions and even giving employment to workers coming from disadvantaged backgrounds [21]. Such analysis must also include calculation of the larger social benefit associated with the potential for the double-fortified salt to additionally and simultaneously reduce the impacts of iodine deficiency in the population [5]. Note, additionally, that the present salt enterprise represents the first attempt to build industrial-scale capacity on the island for processing large volumes of salt to meet various local needs, which apart from providing a market for local raw salt producers can also act as means to significantly stabilize the price of salt sold in the local markets. These benefits, however, must be contrasted against potential adverse effects, such as domination of the market by the growing enterprise, requiring an analysis of how best to compensate for such loses. Recent developments in applying Social Returns on Investment (SROI) approaches for comparing the full monetized social costs of a program with the full monetized social benefits of achieving a health outcome (or set of outcomes) may offer a means for undertaking this fuller analysis [22,51].

We have also used rough first calculations of the population coverages that could be obtained with the expansion of salt production in the present cost-effectiveness modelling study. Field studies to assess the actual household coverage achieved through the enterprise will be critical for not only more realistically quantifying its effectiveness for accomplishing LF transmission interruption in a community, but also for identifying better marketing strategies to achieve good population coverage.

In conclusion, we have presented an economic and financial analysis of the Haitian salt social enterprise, which indicates that it may present a sustainable and socially-responsible strategy for aiding the elimination of LF via the marketing of DEC-medicated salt in settings facing fiscal, infrastructural and logistical challenges for delivering tablet-based elimination programs. Results from the break-even projections carried out in this study indicate that the strategy may even have the potential to achieve zero financial costs to a provider once it attains profitability (i.e., results in a positive cash-flow). This study further has shown that the Haitian salt enterprise may have already reached production and sales levels that could result in the coverage of the Léogâne study population at proportions sufficient enough to break LF transmission. Finally, our simulation-based cost-effectiveness study has indicated that because of: 1) increasing revenue from the sale of the DEC salt obtained over time, 2) expansion of its delivery in the population, and 3) the effect of continuous consumption of the drug, even at low daily per capita dosages, leading to a cumulative impact on the survival of worms and mf higher than that afforded by the higher-dosed annual MDA treatment [18], the delivery of DEC through the present Haiti salt enterprise may represent a dramatically more cost-effective option than annual DEC tablet-based MDA for accomplishing LF elimination. While these are encouraging first results and highlight both the economic viability and social effectiveness of using a salt enterprise in the fight against LF, it is clear that efforts to more fully quantify the social value and strategies for developing similar salt social enterprises elsewhere in other endemic settings with different market structures than those of Haiti are now required if the comparative or joint utility of the approach among the current arsenal of LF intervention strategies is to be fully appraised and understood. We note that the means by which the global iodization of edible salt has been accomplished successfully over the past two decades may offer a particularly apt model for building and sustaining the present intervention globally, and suggest that similar tactics used in that program based on introducing DEC medication into prevailing salt production and distribution systems, collaboration with the national and regional salt industries, and engagement with the government sector, civic society and the general public [52], could also make the universal deployment of DEC-medicated salt eminently possible. With less than three years remaining for meeting the initial 2020 target set by WHO for accomplishing the global elimination of LF, the present results indicate that these appraisals and development of policies and strategies for delivery of DEC-salt, either via deployment of similarly-fashioned salt enterprises, such as the present, or through mobilization of existing salt industries, perhaps along with health system-led MDA and vector-control programs, in socially-challenging environments, like Haiti, would improve our current efforts for meeting this laudable but exacting goal successfully.

## Supporting information

S1 Supporting Information Lymphatic Filariasis model descriptions. (DOCX)

## References

[1] World Health Organization. Global programme to eliminate lymphatic filariasis. WHO. Wkly Epidemiol Rec. 2007;82(42):361–380. 17948605

[2] World Health Organization. Monitoring and epidemiological assessment of mass drug administration in the global programme to eliminate lymphatic filariasis: a manual for national elimination programmes. WHO, 2011.

[3] World Health Organization. Elimination of Lymphatic Filariasis as a public health problem-Resolution of the Executive Board of the WHO (WHA50.29) Fifth World Health Assembly, Geneva, Switzerland WHO, 1997.

[4] Bockarie MJ, Kelly-Hope LA, Rebollo M, Molyneux DH. Preventive chemotherapy as a strategy for elimination of neglected tropical parasitic diseases: endgame challenges. Phil Trans R Soc B. 2013;368(1623):20120144 10.1098/rstb.2012.0144 23798692 PMC3720042

[5] Freeman AR, Lammie PJ, Houston R, LaPointe MD, Streit TG, Jooste PL, et al A community-based trial for the control of lymphatic filariasis and iodine deficiency using salt fortified with diethylcarbamazine and iodine. Am J Trop Med Hyg. 2001;65(6):865–871. 10.4269/ajtmh.2001.65.865 11791989

[6] Klepac P, Metcalf CJE, McLean AR, Hampson K. Towards the endgame and beyond: complexities and challenges for the elimination of infectious diseases. Phil Trans R Soc B. 2013;368(1623): 20120137 10.1098/rstb.2012.0137 23798686 PMC3720036

[7] Lammie P, Milner T, Houston R. Unfulfilled potential. Using diethylcarbamazine-fortified salt to eliminate lymphatic filariasis. Bull World Health Organ. 2007;85:545–549. 10.2471/BLT.06.034108 17768503 PMC2636360

[8] Oscar R, Lemoine JF, Direny AN, Desir L, de Rochars VEMB, Poirier MJ, et al Haiti National Program for the Elimination of Lymphatic Filariasis: A Model of Success in the Face of Adversity. PLoS Negl Trop Dis. 2014;8(7):e2915 10.1371/journal.pntd.0002915 25032697 PMC4102456

[9] Irvine MA, Stolk WA, Smith ME, Subramanian S, Singh BK, Weil GJ, et al Effectiveness of a triple-drug regimen for global elimination of lymphatic filariasis: a modelling study. Lancet Infect Dis. 2017;17(4):451–458. 10.1016/S1473-3099(16)30467-4 28012943

[10] Kastner RJ, Stone CM, Steinmann P, Tanner M, Tediosi F. What is needed to eradicate lymphatic filariasis? A model-based assessment on the impact of scaling up mass drug administration programs. PLoS Negl Trop Dis. 2015;9(10):e0004147 10.1371/journal.pntd.0004147 26451729 PMC4599939

[11] Michael E, Singh BK. Heterogeneous dynamics, robustness/fragility trade-offs, and the eradication of the macroparasitic disease, lymphatic filariasis. BMC Med. 2016;14(1):14. 26822124 10.1186/s12916-016-0557-y PMC4731922

[12] Fan P. Eradication of bancroftian filariasis by diethylcarbamazine-medicated common salt on Little Kinmen (Liehyu District), Kinmen (Quemoy) Islands, Republic of China. Ann Trop Med Parasitol. 1990;84(1):25–33. 10.1080/00034983.1990.11812430 2184787

[13] Meyrowitsch D, Simonsen P, Makunde W. Bancroftian filariasis: analysis of infection and disease in five endemic communities of north-eastern Tanzania. Ann Trop Med Parasitol. 1995;89(6):653–663. 10.1080/00034983.1995.11812999 8745940

[14] Meyrowitsch D, Simonsen P, Makunde W. Mass DEC chemotherapy for control of bancroftian filariasis: comparative efficacy of four strategies two years after start of treatment. Trans R Soc Trop Med Hyg. 1996;90(4):423–428. 10.1016/s0035-9203(96)90534-9 8882196

[15] Narasimham M, Sharma S, Sundaram R, Reddy G, Raina V, Sambasivam V, et al Control of bancroftian filariasis by diethylcarbamazine medicated common salt in Karaikal, Pondicherry, India. J Infect Dis. 1989;21(3):157–170. 2693530

[16] Rao C, Panduranga R, Russel S. Control of bancroftian filariasis with common salt medicated with diethylcarbamazine in Lakshadweep. Indian J Med Res. 1981;73:865–873.

[17] Reddy GS, Venkateswaralu N. Mass administration of DEC-medicated salt for filariasis control in the endemic population of Karaikal, south India: implementation and impact assessment. Bull World Health Organ. 1996;74(1):85 8653820 PMC2486855

[18] Smith ME, Singh BK, Michael E. Assessing endgame strategies for the elimination of lymphatic filariasis: A model-based evaluation of the impact of DEC-medicated salt. Sci Rep. 2017;7(1):7386 10.1038/s41598-017-07782-9 28785097 PMC5547057

[19] World Health Organization. Global programme to eliminate Lymphatic Filariasis. WHO. Wkly Epidemiol Rec. 2010;365–372. 20853547

[20] Grady CA, De Rochars MB, Direny AN, Orelus JN, Wendt J, Radday J, et al Endpoints for lymphatic filariasis programs. Emerg Infect Dis. 2007;13(4):608 10.3201/eid1304.061063 17553278 PMC2725965

[21] Bagnoli L, Megali C. Measuring performance in social enterprises. Nonprofit Volunt Sect Q. 2011;40(1):149–165.

[22] Millar R, Hall K. Social return on investment (SROI) and performance measurement: The opportunities and barriers for social enterprises in health and social care. Public Adm Rev. 2013;15(6):923–941.

[23] Yang CL, Huang RH, Lee YC. Building a performance assessment model for social enterprises-views on social value creation. Sci J Bus Manag. 2014;2(1):1–9.

[24] Kang HY, Schoenung JM. Economic analysis of electronic waste recycling: modeling the cost and revenue of a materials recovery facility in California. Environ Sci Technol. 2006;40(5):1672–1680. 16568786 10.1021/es0503783

[25] Collis J, Hussey R. Break-even Analysis In: Cost and Management Accounting. London: Springer; 1999 p.129–139.

[26] Bierman H Jr, Smidt S. The capital budgeting decision: economic analysis of investment projects. 9th ed. London: Routledge; 2012.

[27] Samonas M. Financial Forecasting, Analysis, and Modelling: A Framework for Long-term Forecasting New York: John Wiley & Sons; 2015.

[28] Boyd A, Won KY, McClintock SK, Donovan CV, Laney SJ, Williams SA, et al A community-based study of factors associated with continuing transmission of lymphatic filariasis in Leogane, Haiti. PLoS Negl Trop Dis. 2010;4(3):e640 10.1371/journal.pntd.0000640 20351776 PMC2843627

[29] De Rochars MB, Kanjilal S, Direny AN, Radday J, Lafontant JG, Mathieu E, et al The Leogane, Haiti demonstration project: decreased microfilaremia and program costs after three years of mass drug administration. Am J Trop Med Hyg. 2005;73(5):888–894. 16282299

[30] Mathieu E, Deming M, Lammie PJ, McLaughlin SI, Beach MJ, Deodat DJ, et al Comparison of methods for estimating drug coverage for filariasis elimination, Leogane Commune, Haiti. Trans R Soc Trop Med Hyg. 2003;97(5):501–505. 10.1016/s0035-9203(03)80006-8 15307410

[31] Mathieu E, Direny AN, De Rochars MB, Streit TG, Addiss DG, Lammie PJ. Participation in three consecutive mass drug administrations in Leogane, Haiti. Trop Med Int Health. 2006;11(6):862–868. 10.1111/j.1365-3156.2006.01626.x 16772008

[32] Michael E, Malecela MN, Zervos M, Kazura JW. Global eradication of lymphatic filariasis: the value of chronic disease control in parasite elimination programmes. PLoS One. 2008;3(8):e2936 10.1371/journal.pone.0002936 18698350 PMC2490717

[33] Michael E, Malecela-Lazaro MN, Maegga BT, Fischer P, Kazura JW. Mathematical models and lymphatic filariasis control: monitoring and evaluating interventions. Trends Parasitol. 2006;22(11):529–535. 10.1016/j.pt.2006.08.011 16971182

[34] Michael E, Malecela-Lazaro MN, Simonsen PE, Pedersen EM, Barker G, Kumar A, et al Mathematical modelling and the control of lymphatic filariasis. Lancet Infect Dis. 2004;4(4):223–234. 10.1016/S1473-3099(04)00973-9 15050941

[35] Singh BK, Bockarie MJ, Gambhir M, Siba PM, Tisch DJ, Kazura J, et al Sequential modelling of the effects of mass drug treatments on anopheline-mediated lymphatic filariasis infection in Papua New Guinea. PLoS One. 2013;8(6):e67004 10.1371/journal.pone.0067004 23826185 PMC3691263

[36] Singh BK, Michael E. Bayesian calibration of simulation models for supporting management of the elimination of the macroparasitic disease, lymphatic filariasis. Parasit Vectors. 2015;8(1):522. 26490350 10.1186/s13071-015-1132-7 PMC4618871

[37] Michael E, Singh BK, Mayala BK, Smith ME, Hampton S, Nabrzyski J. Continental-scale, data-driven predictive assessment of eliminating the vector-borne disease, lymphatic filariasis, in sub-Saharan Africa by 2020. BMC Med. 2017;15(1):176 10.1186/s12916-017-0933-2 28950862 PMC5615442

[38] Goldman AS, Brady MA, Direny A, Desir L, Oscard R, Vely J-F, et al Costs of integrated mass drug administration for neglected tropical diseases in Haiti. Am J Trop Med Hyg. 2011;85(5):826–833. 10.4269/ajtmh.2011.10-0635 22049035 PMC3205627

[39] DSDS: Population Totale, Population de 18 ans et Plus Menages et Densites Estimes en 2015. Institut Haïtien de Statistique et d’Informatique (IHSI), Minstere de L’Economie et des Finances (MEF), Republique d’Haiti, 2015.

[40] USAID: The Haitian sea salt industry: a commercialisation strategy. TechnoServe, March 2012.

[41] Baltussen RM, Adam T, Tan-Torres Edejer T, Hutubessy RC, Acharya A, Evans DB, et al Making choices in health: WHO guide to cost-effectiveness analysis. WHO, Geneva 2003.

[42] Guyatt H. Different approaches to modelling the cost-effectiveness of schistosomiasis control. Mem Inst Oswaldo Cruz. 1998;93:75–84. 10.1590/s0074-02761998000700010 9921326

[43] Hall A, Horton S, de Silva N. The costs and cost-effectiveness of mass treatment for intestinal nematode worm infections using different treatment thresholds. PLoS Negl Trop Dis. 2009;3(3):e402 10.1371/journal.pntd.0000402 19333371 PMC2657832

[44] Kim YE, Sicuri E, Tediosi F. Financial and economic costs of the elimination and eradication of onchocerciasis (River Blindness) in Africa. PLoS Negl Trop Dis. 2015;9(9):e0004056 10.1371/journal.pntd.0004056 26360917 PMC4567329

[45] Lee BY, Bartsch SM, Gorham KM. Economic and financial evaluation of neglected tropical diseases. Adv Parasitol. 2015;87:329–417. 10.1016/bs.apar.2015.01.002 25765199

[46] Lo NC, Bogoch II, Blackburn BG, Raso G, N'Goran EK, Coulibaly JT, et al Comparison of community-wide, integrated mass drug administration strategies for schistosomiasis and soil-transmitted helminthiasis: a cost-effectiveness modelling study. Lancet Glob Health. 2015;3(10):e629–e638. 10.1016/S2214-109X(15)00047-9 26385302

[47] Turner HC, Truscott JE, Fleming FM, Hollingsworth TD, Brooker SJ, Anderson RM. Cost-effectiveness of scaling up mass drug administration for the control of soil-transmitted helminths: a comparison of cost function and constant costs analyses. Lancet Infect Dis. 2016;16(7):838–846. 10.1016/S1473-3099(15)00268-6 26897109

[48] Gedge LM, Bettis AA, Bradley MH, Hollingsworth TD, Turner HC. Economic evaluations of lymphatic filariasis interventions: a systematic review and research needs. Parasit Vectors. 2018;11(1):75 10.1186/s13071-018-2616-z 29391042 PMC5793442

[49] Cordes JJ. Using cost-benefit analysis and social return on investment to evaluate the impact of social enterprise: Promises, implementation, and limitations. Eval Program Plann. 2017;64:98–104. 10.1016/j.evalprogplan.2016.11.008 28011094

[50] Manetti G. The role of blended value accounting in the evaluation of socio-economic impact of social enterprises. Voluntas. 2014;25(2):443–464.

[51] Banke-Thomas AO, Madaj B, Charles A, van den Broek N. Social Return on Investment (SROI) methodology to account for value for money of public health interventions: a systematic review. BMC Pub Health. 2015;15(1):582. 26099274 10.1186/s12889-015-1935-7 PMC4477315

[52] Mannar MV. Making salt iodization truly universal by 2020. IDD Newsl. 2014;42:12–15.

