# Supplementary material for: Economic performance and cost-effectiveness of using a DEC-salt social enterprise for eliminating the major neglected tropical disease, lymphatic filariasis
Source: PLoS Negl Trop Dis. 2019 Jul 1;13(7):e0007094. doi: 10.1371/journal.pntd.0007094 (PMC6625731; doi:10.1371/journal.pntd.0007094)
Supplement: S1 Supporting Information — (DOCX) [file pntd.0007094.s001.docx]

# S1 Supporting Information. Lymphatic filariasis model descriptions

EPIFIL model description and methods

## The mathematical model of LF transmission dynamics

We employed a genus specific mosquito-vectored transmission model of LF to carry out the modelling work in this study [1-7]. Briefly, the state variables of this hybrid coupled partial differential and differential equation model vary over age (*a*) and/or time (*t*), representing changes in the pre-patent worm burden per human host$(P(a,t)),$adult worm burden per human host$(W(a,t)),$the microfilariae (mf) level in the human host modified to reflect infection detection in a 1 mL blood sample$(M(a,t)),$the average number of infective L3 larval stages per mosquito (*L*), and a measure of immunity$(I(a,t))$developed by human hosts against L3 larvae. The state equations comprising this model are:

$$\begin{matrix} \frac{\partial P}{\partial t}+\frac{\partial P}{\partial a}=\lambda\frac{V}{H} h(a)\Omega(a,t)-\mu P(a,t)-\lambda\frac{V}{H} h(a)\Omega(a,t-\tau)\zeta\\ \frac{\partial W}{\partial t}+\frac{\partial W}{\partial a}=\lambda\frac{V}{H} h\left( a \right)\Omega\left( a,t-\tau\right)\zeta-\mu W\left( a,t \right) \\ \begin{matrix} \frac{\partial M}{\partial t}+\frac{\partial M}{\partial a}=\alpha s\phi\left[ w\left( a,t \right),k \right]W\left( a,t \right)-\gamma M\left( a,t \right) \\ \frac{\partial I}{\partial t}+\frac{\partial I}{\partial a}=W_{T}\left( a,t \right)-\delta I\left( a,t \right) \\ \begin{matrix} \\ \frac{dL}{dt}=\lambda\kappa g\int\pi\left( a \right)\left( 1-f\left[ M\left( a,t \right) \right] \right)da-\left( \sigma+\lambda\psi_{1} \right)L \\ L^{*}=\frac{\lambda\kappa g\int\pi(a)(1-f\left[ M\left( a,t \right) \right)da}{\sigma+\lambda\psi_{1}} \end{matrix} \end{matrix} \end{matrix}$$

The above equations involve partial derivatives of four state variables (*P* - pre-patent worm load; *W* - adult worm load; *M* - microfilaria intensity; and *I* - immunity to acquiring new infection due to the pre-existing total worm load where *W_T_ = W(a,t) + P(a,t)*). Given the faster time scale of infection dynamics in the vector compared to the human host, the infective L3-stage larval density in mosquito population is modelled by an ordinary differential equation essentially reflecting the significantly faster time-scale of the infection dynamics in the vector hosts. This allows us to make the simplifying assumption that the density of infective stage larvae in the vector population reaches a dynamic equilibrium (denoted by *L^*^*) rapidly [1,2,5,8,9]. This basic coupled immigration-death structure of the model as well as its recent extensions has been extensively discussed previously [1-3,5,8,9]. The effects of worm patency are captured by considering that at any time *t*, human individuals of age less than or equal to the pre-patency period, *τ*, will have no adult worms or mf, and the rate at which pre-patent worms survive to become adult worms in these individuals at *a > τ* is given by $\zeta=\exp(-\mu\tau)$. The term $f(M)$enables us to account for the different establishment and development rates of the incoming L3-stage larvae as a function of mf distribution in the host population depending on the genus of mosquito vectors as expressed below:

$f[M(a,t)]=\left[\frac{2}{\left[1+\frac{M(a,t)}{k}\left(1-\exp\left[-\frac{r}{\kappa}\right]\right)\right]^{k}}-\frac{1}{\left[1+\frac{M(a,t)}{k}\left(1-\exp\left[-\frac{2r}{\kappa}\right]\right)\right]^{k}}\right]$ for mosquitoes of *Anopheline* genus;

$f[M(a,t)]=\left(1+\frac{M(a,t)}{k}\left(1-\exp\left[-\frac{r}{\kappa}\right]\right)\right)^{-k}$for mosquitoes of *Culicine* genus.

In the above, $k[=k_{0}+k_{Lin}M]$ is the shape parameter of the negative binomial distribution on the mf uptake whereas r and are respectively the rate of initial increase and the maximum level of L3 larvae. See Table 1 for the description of all the model parameters and functions.

**Table A**  - **Description of EPIFIL model parameters and functions.**

| **Parameter** | **Definition (*units*)** | **Range** | **Refs** |
| --- | --- | --- | --- |
| ***λ*** | Number of bites per mosquito (*per month*) | [5, 15] | 1, 2, 5, 10, 11 |
| ***τ*** | Pre-patency period | [1, 9] | 12 |
| ***s*** | Proportion of female worms | 0.5 | - |
| ***μ*** | The worm mortality rate (*per month*) | [0.008, 0.018] | 1, 2, 5, 13-16 |
| ***α*** | Production rate of microfilariae per worm (*per month*) | [0.25, 1.5] | 1, 2, 5, 17 |
| ***γ*** | The death rate of the microfilariae (*per month*) | [0.08, 0.12] | 1, 5, 15, 17 |
| ***g*** | Proportion of mosquitoes which pick up infection when biting an infected host | [0.251, 0.485] | 1, 5, 18 |
| ***κ*** | Maximum level of L3 given mf density | [3, 5] | 1, 5 |
| ***k_0_*** | The basic location parameter of negative binomial distribution used in aggregation parameter  ($k=k_{0}+k_{Lin}M$) | [0.000036, 0.000775] | 1, 5, 19, 20 |
| ***δ*** | Immunity waning rate (*per month*) | [0.001, 0.01] | 1, 5 |
| ***V/H*** | Ratio of number of vector to hosts | $MBR^{\#}/\lambda$ | data |
| ***k_Lin_*** | The linear rate of increase in the aggregation parameter defined above | [0.00000024, 0.282] | 1, 5, 19, 20 |
| ***σ*** | Death rate of mosquitoes (*per month*) | [1.5, 8.5] | 1, 5, 20 |
| ***ψ_1_*** | Proportion of L3 leaving mosquito per bite | [0.1, 0.8] | 17 |
| ***ψ_2_*** | The establishment rate^1^ | [0.00003, 0.00364] | 1, 2, 5, 21 |
| ***H_Lin_*** | A threshold value used in *h(a)* to adjust the rate at which individuals of age *a* are bitten: linear rise from 0 at age zero to 1 at age *H_Lin_* in years.$h(a)=a/H_{Lin}\text{ for }a<H_{Lin};h(a)=1\text{ for }a\geq H_{Lin}$ | [240, 360] months | 1, 5, 9 |
| ***r*** | Gradient of mf uptake^2^ | [0.04, 0.25] | 1, 5 |
| ***c*** | Strength of acquired immunity | [0.015, 0.025] | 1, 5 |
| ***I_C_*** | Strength of immunosuppression^3^ | [0.5, 5.5] | 1, 5 |
| ***S_C_*** | Slope of immunosuppression function^4^  (*per worm/month*) | [0.01, 0.20] | 1, 5 |
| ***Intervention-related parameters*** | | | |
| ***ω*** | Worm killing efficacy of drug (instantaneous) | dependent on drug regimen | 3 |
| ***ε*** | Microfilariae killing efficacy of drug (instantaneous) | dependent on drug regimen | 3 |
| ***δ_reduc_*** | Reduction in the worm’s fecundity over a period of time *p* due to drug | dependent on drug regimen | 3 |
| ***p*** | A time period during which the drug remains efficacious in reducing the fecundity of the surviving adult worms | dependent on drug regimen | 3 |
| ***C*** | Percentage of the population administered the drug | data | data |
| ***MBR_VC_*** | Vector control (VC) modifies $V/H$($=MBR/\lambda$) where  $MBR_{VC}=MBR_{0}\exp[a_{1}t]$, with$a_{1}<0$for $\forall t$when VC is implemented, otherwise$a_{1}>0$. | data and estimates | 19, 20 |
| **Description** | **Mathematical expressions of the functions** | **Parameters** |  |
| Probability that an individual is of age *a* ***π(a)*** | $\pi(a)=A_{0}\exp[-B_{0}a]$ | Human age *a* in month, *A_0_* and *B_0_* estimated from country demographic data | 1, 5, 9 |
| Larvae establishment rate (modified by acquired immunity) ***Ω(a,t)*** | $L^{*}\psi_{1}\psi_{2}g_{1}(I)g_{2}(W_{T})$ | $\psi_{1}$- proportion of L3 leaving mosquito per bite; $\psi_{2}$- the establishment rate^1^ | - |
| Adult worm mating probability ***ϕ(W,k)*** | $1-\left(1+\frac{W}{2k}\right)^{-(1+k)}$ | *k* – negative binomial aggregation parameter | 2, 5, 22 |
| Immunity to larval establishment ***g_1_(I)*** | $\frac{1}{1+cI}$ | *c* – strength of immunity to larval establishment | 1, 5 |
| Host immunosuppression  ***g_2_(W_T_)*** | $\frac{1+I_{C}S_{C}W_{T}}{1+S_{C}W_{T}}$ | *I_C_* – strength of immunosuppression;  *S_C_* – slope of immunosuppression | 1, 5 |

^1^The proportion of L3-stage larvae infecting human hosts that survive to develop into adult worms [2].

^2^The gradient of mf uptake *r* is a measure of the initial increase in the infective L3 larvae uptake by vector as *M* increases from 0 [2,9].

^3^ The facilitated establishment rate of adult worms due to parasite-induced immunosuppression in a heavily infected human host

^4^ The initial rate of increase by which the strength of immunosuppression is achieved as *W* increases from 0 [23].

^#^Note MBR (monthly biting rate) serves as an input to initialize the model, measured as mosquito bites per person per month, the value of which may be obtained from entomological surveys conducted in study sites. In the absence of the observed MBR value, the model has been adapted to estimate it from the community-level mf prevalence data.

**Model calibration to baseline infection data using Bayesian Melding**

We used a data-model assimilation technique based on the Bayesian Melding (BM) algorithm to calibrate and identify the localized LF transmission models applicable to our study site [4-7,26]. This was done by “melding” the observed baseline mf data from Léogâne commune [24,25] with model-generated outputs in order to learn an ensemble of models describing the localized parasite transmission dynamics in this setting. In brief, we first used the known ranges of parameter values to generate distributions of parameter priors. We then randomly sampled from these prior distributions to generate 200,000 parameter vectors, which are used along a range of MBR values to generate model outputs. The model outputs are then melded with data on the mf prevalence age profile, by calculating binomial log-likelihoods of each simulated model for the data. The respective likelihood values are then used to assign weights to each model. In the resampling step of the BM method, 500 draws with replacement are performed selecting from the initial pool of 200,000 models with probabilities proportional to their relative weights. This step generates the most likely model outputs and thus the most likely parameter vectors. The observed overall community mf prevalence (15.5%) recorded in Léogâne [24,25] was translated into theoretical age infection profiles using: 1) the national demographic profile for Haiti, and 2) by conversion of the community-level mf prevalence to reflect either a plateau or concave or high plateau/linear age-infection profile known typically to occur in LF endemic regions [26]. The derived age-prevalence infection data were then used in the model fitting procedures described above, effectively allowing the integration of partially observed data into the model.

## Modeling intervention by mass drug administration

Intervention by mass drug administration was modeled based on the assumption that anti-filarial treatment with a combination drug regimen acts by killing certain fractions of the populations of adult worms and microfilariae instantly after the drug administration. These effects are incorporated into the basic model by calculating the population sizes of worms and microfilariae as follows:

$$\left.\begin{matrix} P(a,t+dt)=(1-\omega C)P(a,t) \\ W(a,t+dt)=(1-\omega C)W(a,t) \\ M(a,t+dt)=(1-\varepsilon C)M(a,t) \end{matrix}\right\} \text{ at time }t=T_{MDA_{i}}$$

where *dt* is a short time period since the *i*th MDA was administered. During this short time interval, a given proportion of adult worms and microfilariae are instantly removed. The parameters *ω* and *ε* are drug killing efficacy rates for the two life stages of the parasite while the parameter *C* represents the MDA coverage. Apart from instantaneous killing of microfilariae, the drug continues to kill the newly reproduced mf by any surviving adult worms at a rate *δ_reduc_* for a period of time, *p* for which the newly produced mf continue to be killed after the i th administration of the drug at time *T_MDAi_*. We model this effect as follows:

where s is the worm sex ratio, α is the mf production rate, ϕ is the worm mating probability, and γ is the mf death rate (Table 1). We simulated LF intervention by running the model with fixed values of *ω, ε*, *δ_reduc_*, and *p* (here δ_reduc_ = 1) given in Michael et al [3] for MDA coverage levels given by data. The first MDA round was implemented in the model by affecting the population sizes of worms and microfilariae from the baseline fits, and then the intervention is simulated forward in time for a number of years, with subsequent MDA rounds implemented annually.

## Modeling intervention by DEC-medicated salt (Double-fortified salt)

The use of DEC-medicated salt as a treatment regimen differs from standard mass drug administration (MDA) primarily in that a small dose of DEC is consumed daily as opposed to a larger dose applied once annually. In the case of annual MDA, the use of DEC is modelled by assuming an instantaneous killing effect on mf and worms at the time of ingestion. However, in the case of daily consumption, there is a continuous input of the drug at low concentration on a daily basis, suggesting a more frequent pulsing or spacing of discrete treatments than in the case of annual or biannual MDA. While it is possible to model DEC salt application using continuous functions, here, we approximated the daily drug intake instead as a monthly pulse where impacts on mf and adult worms are aggregated to reflect clearance rates per month. This was done to facilitate consistency with the monthly scale of the other parameters employed in the model, and use of the drug intervention equations described above for simulating annual or biannual MDA [26] but with *T_MDAi_* assumed to be carried out now at monthly intervals rather than annually.

## References

1. Gambhir, M., Michael, E. Complex ecological dynamics and eradicability of the vector borne macroparasitic disease, lymphatic filariasis. PLoS One. 2008;3:e2874.

2. Gambhir, M. Geographic and ecologic heterogeneity in elimination thresholds for the major vector-borne helminthic disease, lymphatic filariasis. BMC Biol. 2010;8(1):22.

3. Michael E, Malecela-Lazaro MN, Simonsen PE, Pedersen EM, Barker G, Kumar A, et al.Mathematical

modelling and the control of lymphatic filariasis. Lancet Infect Dis. 2004;4(4):223-234.

4. Michael E, Malecela-Lazaro MN, Maegga BT, Fischer P, Kazura JW. Mathematical models and lymphatic filariasis control: monitoring and evaluating interventions. Trends Parasitol. 2006;22(11):529-535

5. Singh BK, Bockarie MJ, Gambhir M, Siba PM, Tisch DJ, Kazura J, et al. Sequential modelling

of the effects of mass drug treatments on anopheline-mediated lymphatic filariasis infection in Papua New

Guinea. PLoS One. 2013;8(6):e67004.

6. Singh BK, Michael E. Bayesian calibration of simulation models for supporting management of the elimination of the macroparasitic disease, lymphatic filariasis. Parasit Vectors. 2015;8(1):522.

7. Michael E, Singh BK: Heterogeneous dynamics, robustness/fragility trade-offs, and the eradication of the macroparasitic disease, lymphatic filariasis. BMC Med. 2016;14(1):14.

8. Chan MS, Srividya A, Norman RA, Pani SP, Ramaiah KD, Vanamail P, et al. EPIFIL: a dynamic model of infection and disease in lymphatic filariasis. Am J Trop Med Hyg. 1998;59(4):606-14.

9. Norman RA, Chan MS, Srividya A, Pani SP, Ramaiah KD, Vanamail P, et al. EPIFIL: the development of an age-structured model for describing the transmission dynamics and control of lymphatic filariasis. Epidemiol Infect. 2000;124(3):529-41.

10. Rajagopalan P. Population dynamics of culex pipiens fatigans, the filariasis vector, in pondicherry: influence of climate and environment. Proc Ind Nat Science Acad B. 1980;46:745-752.

11. Subramanian S, Manoharan A, Ramaiah KD, Das PK. Rates of acquisition and loss of Wuchereria bancrofti infection in Culex quinquefasciatus. Am J Trop Med Hyg. 1994;51:244-249.

12. Scott AL. Lymphatic-dwelling filariae. In: Lymphatic filariasis. Singapore: World Scientific; 2000. p. 5-39.

13. Vanamail P, Subramanian S, Das PK, Pani SP, Rajagopalan PK. Estimation of fecundic life span of Wuchereria bancrofti from longitudinal study of human infection in an endemic area of Pondicherry (South India). Ind J Med Res. 1990;91:293-297.

14. Evans DB, Gelband H, Vlassoff C. Social and economic factors and the control of lymphatic filariasis: a review. Acta Trop. 1993;53:1-26.

15. Ottesen E, Ramachandran C. Lymphatic filariasis infection and disease: control strategies. Parasitol Today. 1995;11:129-130.

16. Vanamail P, Ramaiah KD, Pani SP, Das PK, Grenfell BT, Bundy DA. Estimation of the fecund life span of Wuchereria bancrofti in an endemic area. Trans R Soc Trop Med Hyg. 1996;90(2):119-21.

17. Hairston NG, de Meillon B. On the inefficiency of transmission of Wuchereria bancrofti from mosquito to human host. Bull World Health Organ. 1968;38:935-941.

18. Subramanian S, Krishnamoorthy K, Ramaiah KD, Habbema JD, Das PK, Plaisier AP. The relationship between microfilarial load in the human host and uptake and development of Wuchereria bancrofti microfilariae by Culex quinquefasciatus: a study under natural conditions. Parasitology. 1998;116(3):243-55.

19. Subramanian S, Pani S, Das P, Rajagopalan P. Bancroftian filariasis in Pondicherry, south India: 2. Epidemiological evaluation of the effect of vector control. Epidemiol Infect. 1989;103: 693-702.

20. Das PK, Manoharan A, Subramanian S, Ramaiah KD, Pani SP, Rajavel AR, et al. Bancroftian filariasis in Pondicherry, south India–epidemiological impact of recovery of the vector population. Epidemiol Infect. 1992;108(3):483-93.

21. Ho BC, Ewert A. Experimental transmission of filarial larvae in relation to feeding behaviour of the mosquito vectors. Trans R Soc Trop Med Hyg. 1967;61:663-666.

22. May RM. Togetherness among schistosomes: its effects on the dynamics of the infection. Math Biosci. 1977;35:301-343.

23. Duerr H, Dietz K, Eichner M. Determinants of the eradicability of filarial infections: a conceptual approach. Trends Parasitol. 2005;21:88-96.

24. Boyd A, Won KY, McClintock SK, Donovan CV, Laney SJ, Williams SA, et al. A community-based study of factors associated with continuing transmission of lymphatic filariasis in Leogane, Haiti. PLoS Negl Trop Dis. 2010;4(3):e640.

25. De Rochars MB, Kanjilal S, Direny AN, Radday J, Lafontant JG, Mathieu E, et al. The Leogane,

Haiti demonstration project: decreased microfilaremia and program costs after three years of mass drug

administration. Am J Trop Med Hyg. 2005;73(5):888-894.

26. Smith ME, Singh BK, Michael E. Assessing endgame strategies for the elimination of

lymphatic filariasis: A model-based evaluation of the impact of DEC-medicated salt. Sci Rep.

2017;7(1):7386.
